# Characteristics of Malpractice Litigation Involving Pathological Autopsies Themselves in Japan: A Database Analysis

**DOI:** 10.31662/jmaj.2023-0003

**Published:** 2023-09-29

**Authors:** Masayuki Ohira

**Affiliations:** 1 Department of General Internal Medicine and Clinical Laboratory, National Center of Neurology and Psychiatry National Center Hospital, Tokyo, Japan; 2 Jinho Law Office, Tokyo, Japan

**Keywords:** malpractice lawsuits, pathological autopsy, informed consent

## Abstract

**Introduction:**

Following recently increased public awareness, the number of medical malpractice litigation cases in Japan has increased in all fields of health care. A pathological autopsy is important for medical practice but has not yet been subject to much litigation.

**Methods:**

This study presents a review of all civil lawsuits involving pathological autopsies identified in a Japanese database of lawsuits (Westlaw.com). The basic characteristics of cases, the main issues in each case, results, and reasons for the results given by the court were examined.

**Results:**

Over 500 cases were examined, among which four autopsy-related negligence lawsuits were retained for analysis. Judgments in these cases were delivered between February 2000 and February 2017. Two judgments were regarding the same case, which was discussed in two different civil procedures (monetary damages and request to return the specimens of patients to relatives). This included three factual cases, which were all civil. Each case was brought by close relatives, and all defendants were hospitals. The main issues were informed consent and the accuracy of autopsy results in two and one cases, respectively. The issues related to informed consent were the need for informed consent to take a specimen and the scope of informed consent.

**Conclusions:**

This study describes the basic characteristics of malpractice lawsuits related to autopsies. Lawsuits related to pathological autopsies seem to be relatively rare in Japan.

## Introduction

Medical malpractice lawsuits have been filed for every step of daily medical practice. Awareness about the risk of medical errors has been increasing in Japan for several decades, but no research has clarified the characteristics and patterns of errors in the course of pathological autopsies. 

In Japan, hospital autopsies are typically performed when inpatients die, and there is little malpractice litigation related to hospital autopsies intrinsic themselves. However, autopsies usually require informed consent for both their performance and the scope of their target. Various specimens can also be procured during autopsies and kept for further medical research. Therefore, steps related to pathological autopsies may also be the subject of malpractice, such as clinical biopsies in normal medical settings. However, to date, there is no research regarding lawsuits whose main issue is related to pathological autopsy. 

Medical malpractice lawsuits related to pathological autopsies have not been studied. A comprehensive medical error reporting system that is suitable for research purposes does not exist in Japan ^(1)^. However, malpractice research has drawn on information about closed malpractice claims provided by insurers in other countries ^(2), (3)^. Past court decisions are important to assess issues and risks in various clinical settings, including for autopsies in Japan. Past studies on medical malpractice lawsuits in Japan have been limited to analyses of publicly accessible judicial cases obtained using various databases. In this study, the characteristics of malpractice litigation related to pathological autopsies in Japan were examined to identify cautionary points to prevent future conflicts.

## Materials and Methods

I performed a retrospective review of malpractice cases related to autopsies. Cases were identified via a nationwide online database search of legal cases in Japan (https://www.westlawjapan.com/). Westlaw is a comprehensive legal research engine that is used in various types of medical malpractice research in other countries ^(4), (5), (6)^. Westlaw Japan is the Japanese version of Westlaw and provides the largest case database in Japan, covering more than 300,000 cases dating back to the prewar era ^(7)^. On December 31, 2022, cases since 1949 were searched using keywords including “Postmortem Examination and Corpse Preservation Act” (PECP), which is Act No. 204 of 1949 that legalized various autopsies including pathological ones, and “pathological autopsy,” which refers to all autopsies except those required for forensic investigation or administrative requirements by statute in Japan. All cases were screened for date and court, plaintiff, and defendant, results, and main issues by the author, who is certified as a Fellow of the Japanese Society of Internal Medicine and is a licensed Japanese attorney and a member of a law firm in Tokyo, Japan.

## Results

Overall, 25 and 455 cases were detected using “PECP” and “pathological autopsy” as search terms, respectively. All cases were assessed by the author to verify whether the main issues included pathological autopsy. Overall, only nine cases discussed issues related to pathological autopsy. In those nine cases, one was regarding the proper and respectful handling of a corpse after an autopsy by a funeral company, one case discussed the requirement to disclose the autopsy report, and three cases discussed the need for a pathological autopsy when one had not been performed. Those five cases were excluded because they were not related to the pathological autopsy procedure itself and the implementation of pathological autopsy in Japan is usually decided without a pathologist. Therefore, only four cases were included in this analysis.

The judgments in the four autopsy-related negligence lawsuits were delivered between February 2000 and February 2017 (Table 1). In Case 1, the parents sued the hospital because its doctors took specimens from the corpse of their child without any consent. Two lawsuits (Cases 2 and 3) depended on the same factual case but involved two different civil procedures (regarding monetary damages and the return of pathological specimens). In these cases, the bone, and bone marrow were taken during the autopsy and were retained by the hospital. The bereaved family claimed that they had never agreed to donate those specimens and sued for their return (Case 2) and monetary damages due to the infliction of emotional distress (Case 3). In Case 4, the husband sued the hospital based on pathological misdiagnosis after autopsy because the autopsy results did not correspond with what he believed. All cases were civil cases brought by close relatives: parents (Case 1), children (Cases 2 and 3), and spouse (Case 4). The defendants were a public hospital in Case 1, university hospitals in Cases 2 and 3, and a national hospital in Case 4. The basic facts were the same for Cases 2 and 3, but the results varied. The case involving monetary damages (Case 3) was dismissed, but the request for the return of specimens was approved (Case 2). This division was caused by differences in factual acknowledgment of informed consent. Monetary damages were approved only in Case 1, and the amount was relatively large, 76,646,272 JPY or approximately 56,000 USD. The reason for granting damages was because the death of the patient in Case 1 was affirmed to be caused by medical malpractice and the damage was based on the issue related to not only the pathological autopsy but also the death itself. The main issues were informed consent in three cases (Cases 1, 2, and 3) and the accuracy of the autopsy results (Case 4). The details of those issues and a summary of each court’s decision are shown in Table 2.

**Table 1.** Basic Characteristics of Litigation Related to Pathological Autopsies in Japan.

Case no.	Date of judgment	Name of court	Characteristics of plaintiff (relation to the deceased)	Characteristics of defendant	Conclusion	Approved damage (JPY)
1	Feb 1, 2000	Fukuoka HC, Miyazaki branch	Parents	Public Hospital	Approved	76,646,272
2	Nov 24, 2000	Tokyo DC	Children	University Hospital	Approved	N/A
3	Aug 30, 2001	Tokyo DC	Children	University Hospital	Dismissed	0
4	Feb 5, 2007	Tokyo DC	Spouse	National Hospital	Dismissed	0

DC, District Court; HC, High Court

**Table 2.** Main Issues and Reasons for Decisions in Litigation Related to Pathological Autopsies in Japan.

Case no.	Main issues	Summary of courts’ decision on main issues
1	Taking liver specimen from a corpse without informed consent	Acquiring specimens without proper consent was illegal.
2	The scope of informed consent about bone and bone marrow and duty to return specimens to plaintiff	Proper informed consent was not acquired by defendant, who was required to return specimens such as paraffin-embedded tissue blocks to the plaintiff.
3	Same as Case 3	The defendant was not required to obtain specific consent for keeping bone and bone marrow samples beyond the consent to keep “organs.” The proper informed consent existed, and the specimens’ ownership was therefore considered to move from plaintiff to defendant.
4	Misdiagnosis of pathological condition after anatomical autopsy	The final diagnosis after autopsy was correct, and there was no breach of duty by the hospital.

## Discussion

In this search, only four lawsuits on issues related to pathological autopsy itself were found. Conflicts arising as a direct result of the pathological autopsy procedure are supposedly rare in Japan. The PECP is a statute that allows for the dissection of the body in specific situations, including gross anatomy dissection, and pathological autopsy in medical and dental schools and hospitals. This legislation was enacted in 1949 for the improvement of public health and education and research, and it has conditions for the legal preservation of specimens after autopsies. However, for example, anatomical gifting, such as brain donation to brain banks where specimens are supposed to be procured and accumulated for supplying future researchers, did not exist in Japan at that time. Therefore, the PECP could have gaps regarding those new medical settings because it is outdated, and the PECP may be insufficient to avoid conflicts between patients and medical professionals over pathological autopsies and the donation of specimens. Thus, although malpractice cases are rare, it is useful for medical professionals to analyze cases related to the pathological autopsy procedure.

Despite the rarity of conflicts related to pathological autopsies, two cases were focused on issues related to informed consent. Informed consent has been reported as a possible cause of medical malpractice lawsuits in other areas ^(8)^. As with other medical practices, informed consent provided by patients’ relatives is necessary and important in pathological autopsies. Both proper informed consent and the correct scope of informed consent are crucial because conflicts might arise related to going beyond the perceived scope of informed consent According to these cases, the informed consent of the pathological autopsy should cover a clear scope of the autopsy to avoid misunderstanding and future conflict. Additionally, clear explanations for, and full understanding by bereaved families are required. However, conducting the proper informed consent process can help to avoid future conflicts and secure the development of the pathological autopsy process in Japan.

This study had several limitations. This was a single-institutional retrospective review, and all cases were examined by only one investigator. Thus, there may have been biases affecting the construction of each case.

### Conclusions

This study reported the basic characteristics of medical malpractice lawsuits related to the pathological autopsy procedure in Japan. Cases were rare, but the findings suggest that informed consent could be the main issue in autopsy malpractice cases and other medical malpractice lawsuits. Pathological autopsies rarely cause conflicts between medical professionals and the families of deceased patients. However, focusing on the proper informed consent process might prevent and reduce such conflicts in the future.

## Article Information

### Conflicts of Interest

None

### Sources of Funding

This work was supported by a grant-in-aid (20FC1054) from the Research Committee of Prion Disease and Slow Virus Infection from the Ministry of Health, Labour and Welfare of Japan and AMED (grant number JP21wm0425019).

### Acknowledgement

We thank Melissa Leffler, MBA; and Analisa Avila, MPH, ELS, from Edanz (https://jp.edanz.com/ac) for editing a draft of this manuscript.

### Author Contributions

Masayuki Ohira contributed to the design and implementation of the study, analysis of the results, and writing of the manuscript.

### Approval by Institutional Review Board (IRB)

Not applicable.
